# Divergent evolution of hepatocellular carcinoma genomes in chimpanzees and humans

**DOI:** 10.1093/emph/eoaf038

**Published:** 2025-12-15

**Authors:** Lin Kang, Katarzyna Michalak, Robin Varghese, Ramu Anandakrishnan, Edward J Dick, Zakaria Abd Elmageed, Pawel Michalak

**Affiliations:** Division of Biomedical Affairs and Research, Edward Via College of Osteopathic Medicine, Monroe, LA 71203, USA; Center for One Health Research; VA-MD Regional College of Veterinary Medicine, Blacksburg, VA 24060, USA; College of Pharmacy, University of Louisiana Monroe, Monroe, LA 71203, USA; Division of Biomedical Affairs and Research, Edward Via College of Osteopathic Medicine, Monroe, LA 71203, USA; Center for One Health Research; VA-MD Regional College of Veterinary Medicine, Blacksburg, VA 24060, USA; Division of Biomedical Affairs and Research, Edward Via College of Osteopathic Medicine, Blacksburg, VA 24061, USA; Center for One Health Research; VA-MD Regional College of Veterinary Medicine, Blacksburg, VA 24060, USA; Division of Biomedical Affairs and Research, Edward Via College of Osteopathic Medicine, Blacksburg, VA 24061, USA; Southwest National Primate Research Center, Texas Biomedical Research Institute, San Antonio, TX 78245, USA; Division of Biomedical Affairs and Research, Edward Via College of Osteopathic Medicine, Monroe, LA 71203, USA; Division of Biomedical Affairs and Research, Edward Via College of Osteopathic Medicine, Monroe, LA 71203, USA; Center for One Health Research; VA-MD Regional College of Veterinary Medicine, Blacksburg, VA 24060, USA; Institute of Evolution, University of Haifa, Haifa 3498838, Israel

**Keywords:** comparative cancer genomics, cancer evolution, primate genomics, somatic mutations

## Abstract

**Background and objectives:**

Somatic mutation patterns in cancer remain largely unexplored outside humans, despite their significance for aging and oncogenesis. Chimpanzees (*Pan troglodytes*), sharing >98% genomic similarity with humans, display markedly different cancer spectra. To gain comparative insights into cancer susceptibility and resistance, we sequenced chimpanzee hepatocellular carcinoma (HCC) genomes and analyzed their mutational profiles alongside human counterparts.

**Methodology:**

HCC and matched non-cancerous tissues from five chimpanzees were examined using histopathology, immunohistochemistry (β-catenin, ARID1A, TSC2, FAP, vimentin, TGF-β), whole-genome sequencing (one pair), and whole-exome sequencing (four pairs). Somatic variants were identified with GATK MuTect2, annotated with Ensembl VEP, and analyzed for functional enrichment. Comparative analyses were performed with subsets of human HCC datasets (TCGA, ICGC) including *TSC2*-positive and *TSC2*-negative cases.

**Results:**

Chimpanzee HCCs exhibited histological and immunohistochemical features similar to human tumors but displayed sharply divergent genomic landscapes. Chimpanzee tumors carried significantly higher coding mutation loads (mean 5632 per sample vs. 96–275 in humans). Non-synonymous ***TSC2*** mutations occurred in 80% of chimpanzees, versus ~7% in human HCC, suggesting a species-specific oncogenic pathway linked to the scirrhous subtype. Additional recurrently mutated genes included ***ARID1A*, *FAT1–4*, *TP53*,** and ***FGA***. Despite greater heterogeneity in chimpanzee tumors, humans showed stronger enrichment of non-synonymous single nucleotide variants, implying more intense positive selection. Shared alterations across species involved canonical drivers such as ***TP53*, *CTNNB1*, *FAT4*,** and ***TTN*.**

**Conclusions and implications:**

Chimpanzee HCCs are defined by high mutational burden and frequent ***TSC2*** alterations, contrasting with the more selectively constrained mutation spectrum of human HCC. Divergent evolutionary patterns highlight species-specific oncogenic routes while underscoring conserved pathways. Comparative primate cancer genomics offers novel insights into cancer evolution, biomarkers, and therapeutic targets.

## INTRODUCTION

As a consequence of multicellularity, cancer is a ubiquitous disease among metazoans [1]. Selection pressure for molecular mechanisms of natural cancer resistance is very strong because an animal that develops cancer prematurely will leave less or no progeny. A comparative genomics approach to studying cancer across diverse phylogenetic lineages offers significant insights into alternative variations within and among immune-oncologic pathways, which extend beyond those observed in humans. This can greatly advance the field of precision oncology, including the development of targeted therapies.

This approach applied to whole genome data from various mammalian species has already begun to provide striking examples of genetic novelties contributing to cancer resistance, as well as “Peto’s paradox,” or the lack of correlation between body size and cancer risk among mammalian species [2]. Peto’s paradox goes against the prediction that large animals have more cells in their bodies, tend to live longer, and thus should have a statistically higher risk of developing malignancy [3]. Remarkably, the elephant genome encodes 19 extra copies of the tumor suppressor gene *TP53*, which coincides with a hyperactive TP53 signaling pathway and the evolution of large body size [4, 5]. Whales, including the longest-lived bowhead whale (*Balaena mysticetus*) with its lifespan exceeding 200 years, have evolved an alternative mechanism to cope with increased cancer risk through adaptive changes to DNA repair genes [6]. Species- or lineage-unique genetic innovations related to cancer suppression have been found in many other long-lived and unusually cancer-resistant mammalian species, such as naked mole rat (*Heterocephalus glaber*) [7], blind mole rat (*Spalax ehrenbergi*) [8], Brandt’s bat (*Myotis brandtii*) [9], and possibly even primates [10].

Despite these advances, relatively few studies have examined the evolutionary divergence of cancer-related genes specifically between humans and chimpanzees, the two closely related species with strikingly different cancer susceptibilities. Comparative genomic scans have identified several loci under positive selection in the human lineage, including tumor suppressor and DNA repair genes [10, 11], and revealed structural and regulatory divergence in cancer-associated loci such as *BRCA1* [12], *ELAC2* [13], and *FHIT* [14]. These findings suggest that subtle evolutionary modifications in genes involved in genome stability, cell-cycle control, and DNA repair may contribute to species-specific differences in cancer vulnerability. Moreover, cross-species expression analyses indicate that human-specific amino acid changes and regulatory rewiring in key pathways may alter biochemical and oncogenic properties relative to chimpanzees [15].

In contrast to interspecies germline divergence in cancer-related genes as represented by reference genomes, differences in the landscapes of somatic mutations in cancer are largely unknown outside of our own species. Despite widely variable life histories among mammalian species, including variation of around 30-fold in lifespan and around 40 000-fold in body mass, the somatic mutation load in non-cancerous tissues at the end of lifespan remained relatively constant, varying only by a factor of around three [16]. Oncogenic phenomena are common in mammals and exhibit substantial differences in cancer mortality across major mammalian orders [17]. The phylogenetic distribution of cancer mortality appears to be associated with diet, with carnivorous mammals, particularly those that consume other mammals, experiencing the highest cancer-related mortality [17]. Cancer rates among humans are relatively high and increasing, with the three most common cancer types being skin, lung, and breast carcinomas [1].

In comparison, cancer has been uncommon in nonhuman primates, particularly in chimpanzees (*Pan troglodytes*), although it has become increasingly manifest as primate colonies aged [18, 19]. The most common chimpanzee cancers are uterine leiomyomas, followed by hepatocellular carcinomas (HCCs) and ovarian stromal tumors [18], but to date no attempts have been made to characterize those at the molecular or genomic level and compare them with similar human malignancies. While large-scale screens for somatic mutations in human cancer genomes are currently in routine use, there has been little traction for collecting DNA sequences from non-human primate cancers, mostly due to their rarity. Here we highlight genomic changes related to HCCs and divergent evolution patterns of the cancer genomes in chimpanzees and humans.

Human HCC is the most common type of primary liver cancer and the second leading cause of cancer-related deaths worldwide [20]. Chronic viral hepatitis (Hepatitis B virus/HBV and Hepatitis C virus/HCV) remains a major cause of liver cancers in humans, and the relative prevalence of HCC among captive chimpanzees may be associated with their exposure to HBV and HCV infections, as well as *Schistosoma mansoni* and human immunodeficiency virus (HIV) co-infections [21, 22]. Chimpanzees are the only non-human primates that can develop acute and chronic HBV and HCV infections and associated hepatitis. For this reason, they were considered the most important animal model for viral hepatitis research before a ban on chimpanzee research was enforced [23, 24].

## METHODS

### Chimpanzee samples

This study was conducted in accordance with the Institutional Animal Care and Use Committee (IACUC) guidelines, approved by The Southwest National Primate Research Center, Texas Biomedical Research Institute, San Antonio, TX (IACUC #1516 PT). Five chimpanzees diagnosed with HCC were included in the study (Supplementary Table S1). The average age of the animals was 33.8 ± 9.2 years, with 40% (2/5) being female and 60% (3/5) male. The animals weighed an average of 59.02 ± 5.6 kg before their termination. All animals had confirmed diagnoses of HCC, with two exhibiting metastases to the lungs, and one (20%) additionally showing metastasis to the kidneys and lymph nodes. The liver cancer diagnosis was confirmed microscopically by a pathologist. For each liver cancer sample, kidney tissues from the same animal were collected as normal samples, except in one case of metastasis, where heart tissue was used as the normal sample. One individual tested positive for HBV and HIV, while four tested positive for HCV (Supplementary Table S1).

### Immunostaining of chimp tissues

Formalin-fixed paraffin-embedded liver and its matched tissue blocks of adult chimpanzees were obtained from Texas Biomedical Research Institute, Southwest National Primate Research Center, San Antonio, TX. The tissues provided were examined and confirmed as HCC by a cytopathologist using H&E staining and specific HCC markers (Supplementary Table S2 and Fig. S1). The immunostaining of tissues was performed as per our previous reports [25, 26]. Briefly, chimpanzee tissues were cut into 5 μm thick sections. The tissue slides were de-waxed in a series of xylene solutions and rehydrated in descending dilutions of ethanol. The tissue slides heated for 20 min in EDTA buffer solution at pH 8.0. The tissue slides incubated in 3% peroxide to block endogenous peroxidases activity. After washing and blocking steps, the tissue slides were incubated with anti-β-catenin, anti-Tuberin/TSC2, anti-ARID1A/BAF250A, anti-FAB, anti-Vimentin, and anti- TGF-β primary antibodies for 60 min at 37°C (Cell Signaling Technology, Danvers, MA, USA). Other tissue slides were incubated with PBS and kept as a negative control. Sections were washed and incubated with the appropriate biotinylated secondary antibodies (Dako, Carpinteria, CA, USA), followed by incubation with Avidin DH and biotinylated horseradish peroxidase H complex according to the standard protocol (Vector Laboratories, Burlingame, CA, USA). After washing, the reaction was visualized with 3,3′-Diaminobenzidine. Tissue sections were counterstained with hematoxylin, dehydrated, and cleared in xylene then mounted in mounting medium. The developed protein staining was acquired using an AmScope microscope with camera (AmScope, Irvine CA). The tissue slides were blindly examined and expressed as histochemical score (HCS) as reported [27]. The immunostaining of each protein was considered negative when HCS is 0, weak expression (1–2), moderate expression (3–5), and strong expression (6–8).

### DNA extraction and sequencing

DNA was extracted from 10 FFPE tissues using the QIAamp FFPE Advanced kit (Cat# 56704, Qiagen, Germantown, MD) following the manufacturer’s instructions. The quantity of DNA was assessed using Qubit 4 Fluorometer (ThermoFisher, Waltham, MA), and purity was examined using 2100 Agilent Bioanalyzer (Agilent Technologies, Santa Clara, CA). Approximately 1.2 μg of DNA from each sample was used for sequencing. WGS was performed on one sample pair, while WES was conducted on four sample pairs at Genewiz (South Plainfield, NJ) (Supplementary Table S3). Briefly, the SureSelect Human All Exon V6 panel (Agilent Technologies, Inc.) was utilized for whole-exome capture. Previous studies have demonstrated the feasibility of using human-based capture probes for sequencing the exomes of non-human primates [28–30]. We utilized human-based probes from Agilent (SureSelect Human All Exon V6 panel) to capture exomes from four pairs of chimpanzee samples (tumor and normal tissue from the same individual). Enriched DNA from exome capture was then used to construct libraries, which were sequenced using a paired-end (2 × 150 bp) strategy on an Illumina platform, following Agilent’s protocols for whole-exome capture and library construction. For WGS, TruSeq DNA libraries were prepared and sequenced on the HiSeq platform following Illumina’s protocols, and 2 × 150-bp paired- end reads were generated.

### Somatic variant detection

#### Mapping reads to the chimpanzee reference sequence

The chimpanzee reference genome (version Pan_tro_3.0/GCA_000001515.5), along with gene sequences and corresponding annotations, was downloaded from Ensembl (http://useast.ensembl.org/Pan_troglodytes/Info/Index). Raw reads were quality controlled and filtered using FastqMcf [31]. We obtained an average of 13.97 G base pairs of clean WES data for each sample (Supplementary Table S3). The clean reads were mapped to the reference genome using Burrows–Wheeler aligner (version 0.7.17) [32] with default parameters.

#### Variant calling

Variant identification, including short insertions and deletions (indels), was performed using the Genome Analysis Toolkit [33] (version 4.1.8.1) following the best practices for somatic mutation calling [34, 35]. Specifically, MuTect2 [36] was employed for this purpose. A PoN was created from WGS data of 65 wild-born [37] (European Nucleotide Archive accession # PRJEB15086) and 39 captive-born chimpanzees (European Nucleotide Archive accession # PRJEB39475). These populations were chosen to improve variant calling accuracy by providing a comprehensive baseline of normal variants. Candidate somatic variants were generated using GATK MuTect2 (version 2.2) and subsequently filtered using the “FilterMutectCalls” function for orientation bias (“--ob-priors”) and read contamination (“--contamination-table”). Final variant filtering criteria included a minimum allele frequency of 0.05, a minimum median distance from the end of reads of 4, a minimum number of reads carrying variants required on both forward and reverse strands of 1, and a minimum of 2 unique reads supporting the alternate allele (“--min-allele-fraction 0.05 --min-median-read-position 4 --min-reads-per-strand 1 --unique-alt-read-count 2”).

To address the possibility of FFPE-related artifacts, a secondary pipeline was implemented incorporating duplicate marking with “MarkDuplicates,” base quality score recalibration using “BaseRecalibrator,” and application of recalibration via “ApplyBQSR.” This pipeline used a more stringent filtering strategy requiring a minimum allele frequency of 0.12, a minimum median distance from the end of reads of 8, a minimum of 6 unique reads supporting the alternate allele, and at least 3 variant-supporting reads from both the forward and reverse strands via “FilterMutectCalls” (“--min-allele-fraction 0.12 --min-median-read-position 8 --unique-alt-read-count 6 --min-reads-per-strand 3”). The resulting high-confidence (HQ) variant set from the FFPE-aware pipeline was used for mutation burden and spectrum comparisons discussed in this study.

#### Microsatellite variant detection

SMVs were identified using HipSTR [38] (version: 0.7) with default stutter models (“--def-stutter-model”) and *de novo* allele generation. Candidate microsatellite variants were filtered using the following criteria: posterior probability for the genotype higher than 0.90, fraction of indels in reads mapping to the flanking regions of the microsatellite smaller than 0.15, fraction of reads containing stutter artifacts smaller than 0.15, log10 *P* value for the allele bias test higher than 2, and log10 *P* value for the Fisher strand bias test higher than 2 (“--min-call-qual 0.9 --max-call-flank-indel 0.15 --max-call-stutter 0.15 --min-call-allele-bias -2 --min-call-strand-bias -2”).

#### Variant annotation

All identified variants, including insertions, deletions, and microsatellites, were annotated using the Ensembl Variant Effect Predictor [39] (version: 105.0).

#### Human datasets

Three human datasets were analyzed in this study (Supplementary Table S4 and S5):

1) ICGC Dataset: Five HCC sample pairs with *TSC2* somatic mutation from Calderaro *et al.* [40] (International Cancer Genome Consortium/ICGC sample ID: CHC1210, CHC1597, CHC1604, CHC2111, CHC798; referred to as Set ICGC).

2) TCGA+ Dataset: Five random HCC sample pairs from The Cancer Genome Atlas (TCGA; https://portal.gdc.cancer.gov/) liver HCC project (TCGA-LIHC) with TSC2 somatic mutation (referred to as Set TCGA+).

3) TCGA- Dataset: Five random HCC sample pairs from TCGA-LIHC without *TSC2* somatic mutation (referred to as Set TCGA-). Sequencing data from the human datasets, including both tumor samples and matched normal samples (either blood-derived or solid tissue normals), were processed using the same pipeline as used for the chimpanzee data, with the exception that a PoN was not used for the human samples. Although the original ICGC selection criteria included samples reported to harbor *TSC2* mutations, in our re-analysis using a uniform variant-calling pipeline, the number of ICGC human samples meeting our internal mutation threshold for TSC2 was reduced to four. This discrepancy most likely reflects differences in variant-calling sensitivity, filtering thresholds and read-depth between the original ICGC study and our re-analysis.

#### Gene ontology **enrichment and pathway analysis**

Functional enrichment analyses were conducted by importing the relevant gene lists into DAVID [41] and Reactome [42, 43]. Gene Ontology terms with a Benjamini–Hochberg adjusted *P* value of <0.05 were considered significant.

## RESULTS

We analyzed HCC samples and matching non-cancer samples from five adult chimpanzees through tissue immunostaining, whole genome sequencing (WGS), and whole exome sequencing (WES) in comparison with human HCCs.

### Chimpanzee HCC immunohistology

**Figure 1:**
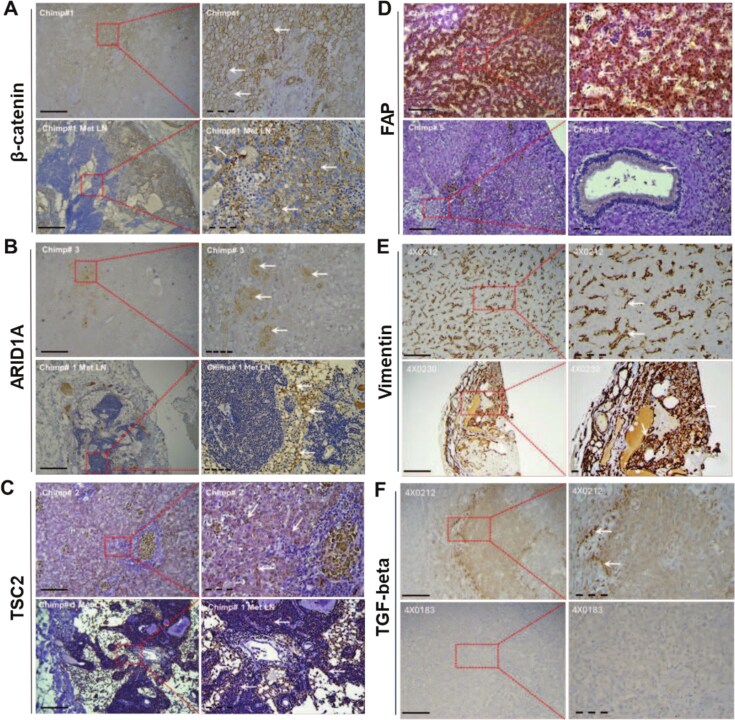
Immunohistochemical analysis of paraffin-embedded chimpanzee tissue specimens; representative photomicrographs of immunohistochemical staining of liver and metastatic lymph node (Met LN) chimpanzee (chimp#1-5) tissues with β-catenin, ARID1A, TSC2, FAP, VIM, and TGF-β antibodies; white arrows indicate positive protein signals; magnifications were 100× (solid line, left panel) and 400× (dashed line, right panel).

HCC is a highly heterogeneous tumor with variable etiology, and therefore molecular markers are commonly used to stratify human patients according to their clinical manifestation and outcomes [44–46]. Expression levels of β-catenin, *ARID1A*, *TSC2*, and *FAP* in paraffin-embedded chimpanzee tissues were assessed through our immunohistochemical analysis (Fig. 1). The HCC samples, including the one metastasized to lymph node (Fig. 1A), were characterized by either high or moderate levels of membranous β-catenin expression (Supplementary Table S2). While normal human hepatocytes do not express β-catenin, it is expressed in HCC cells, accumulating predominantly in the membrane [47]. The protein expression of *ARID1A* was observed in the nucleus and the cytoplasm, with its levels ranging from weak to moderate (Fig. 1B). The AT-rich interactive domain 1A (*ARID1A*, also called *BAF250A*) is a key component of the mammalian SWI/SNF family regulating gene transcription through chromatin remodeling. The nuclear expression of *ARID1A* has been negatively associated with tumor size, pathological grade, tumor recurrence, and survival in human HCC patients [48], while somatic mutations in *ARID1A* have been reported in HCC [49]. Tuberous sclerosis complex 2 (TSC2) is a tumor suppressor gene that plays a critical role in regulating cell growth and proliferation [50]. *TSC2* expression in the chimpanzee samples was mainly cytosolic with high immunostaining in four of the samples, including the metastatic lymph node tissue, and moderate in the remaining two tissue sections (Fig. 1C). Inactivation of *TSC2*, either through mutation or deletion, can lead to uncontrolled cell growth and contribute to tumorigenesis [51]. *TSC2* mutations in human HCC tissues have been associated with more aggressive tumor forms and higher recurrence rate [52]. Fibroblast activation protein (*FAP*) expression was observed in the cytoplasm of cells and 50% of the stained tissues had high protein expression while the rest of tissues had low to moderate staining (Fig. 1D). High *FAP* expression has been reported in HCC and was an independent risk factor for inferior clinical outcome in postsurgical patients [53]. It is also validated as a prognostic biomarker [54]. To further refine the stratification of HCC subtypes, we also used *vimentin* (*VIM*; Fig. 1E) and *TGF-β* (Fig. 1F), typically associated with stromal fibrosis of the HCC tissues and cirrhosis, as well as scirrhous HCC subtype [55]. Stromal cells secrete *TGF-β* which activates fibroblasts and promotes initiation of HCC [56]. Our results show that all chimpanzee HCC tissues (100%) exhibited strong positive staining for VIM, with high signal intensity (Fig. 1E). In contrast, 50% of tissue specimens displayed low to moderate positivity for TGF-β staining (Fig. 1F).

### Somatic mutation landscape in chimpanzee HCC

#### Overview of somatic variants

The sequence mapping rates ranged from 99.77% to 99.95%, with the percentages of paired-end reads uniquely aligned ranging from 94.19% to 99.28% (Supplementary Table S3). This high mapping rate indicates successful exome capture, ensuring the reliability of subsequent variant discovery procedures. A total of 452 277 somatic mutations were identified in the WGS sample (S1), with the majority being single nucleotide variants (SNVs; 317 632 or 70.23%; Supplementary Table S6), and the remainder being indels (110 938 insertions and deletions). In the WES samples (S2, S3, S4, and S5), we found an average of 11 510 SNVs (ranging from 6813 to 14 701) and 13 615 indels (ranging from 6105 to 26 031; Supplementary Table S6). Among the coding variants, we identified 1356 to 2897 non-synonymous mutations (missense and nonsense mutations) and 741 to 5341 frameshift mutations across the five samples (Supplementary Table S7).

#### Functional enrichment of somatic mutations

A total of 120 genes carrying at least one non-synonymous somatic mutation were found in all five chimpanzee HCC samples. Functional enrichment analysis of these 120 genes indicated significant involvement in ATP-dependent microtubule motor activity, dynein chain binding, ATPase activity, motor proteins, and microtubule-based movement (Supplementary Table S8). Additionally, 611 genes bearing non-synonymous somatic mutations were shared in at least four chimpanzee HCC samples. Notably, this included *TSC2* that was associated with the scirrhous subtype of HCC, a rare primary liver tumor with a prevalence in humans ranging from 0.2% to 4.6% [57, 58]. Enrichment analysis of the 611 genes revealed additional significant functions related to ECM-receptor interaction, p53 binding, ATP-binding cassette transporters, and papillomavirus infection (Supplementary Table S9).

#### Comparative analysis of somatic mutations in chimpanzee and human HCC

**Figure 2:**
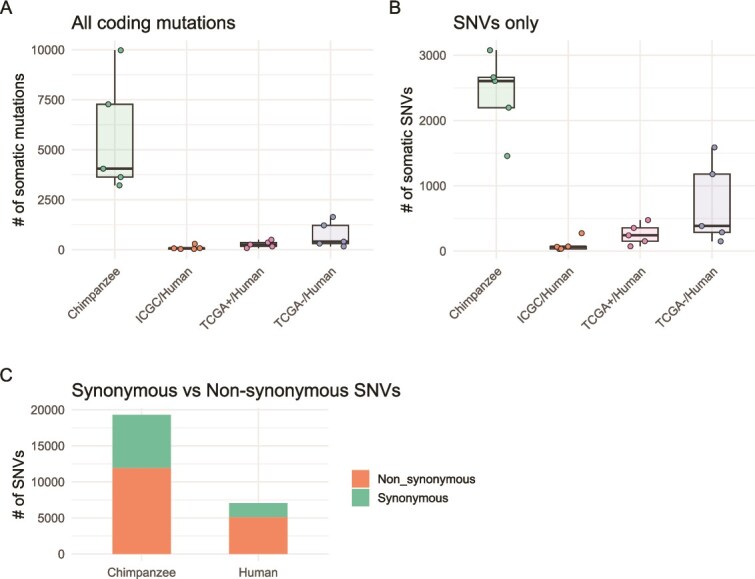
Comparison of somatic variants among chimpanzee HCC samples and three human HCC datasets, all mutations that affect amino acids were included (A) or only SNVs were included (B); (C) synonymous (blue) to non-synonymous mutation (red) ratios in the chimpanzee and human HCC genomes.

Given the significance of *TSC2* mutations in determining the HCC subtype, we compared mutation patterns between chimpanzees and humans using two datasets (TCGA+ & ICGC) of human HCC samples bearing mutant *TSC2* and one set (TCGA-) of human HCC samples without mutant *TSC2* as a control group. Each human dataset contained five sample pairs, randomly selected to match the limited sample size of the chimpanzee dataset. Only coding region mutations resulting in amino acid changes were included in the comparison. On average per sample, 5632 mutations were found in chimpanzee samples, while 275 mutations were found in TCGA+ samples and 96 mutations were found in ICGC samples (Fig. 2A), despite comparable sequencing data amounts across datasets (average of 10.56 GB for human datasets versus 13.97 GB for chimpanzees; Supplementary Table S4). Mutant allele frequency analysis showed that the vast majority of mutations exhibited frequencies below 40% across all four datasets, with a skew toward lower-frequency variants (Supplementary Fig. S2). This distribution suggests a substantial degree of intra-tumor heterogeneity in both chimpanzee and human HCC samples. The predominance of low-frequency variants may partially reflect differences in tumor cellularity and sample purity, factors that are commonly encountered in tumor sequencing studies. SNVs accounted for an average of 47.78% of all mutations in chimpanzee samples, compared to 92.31% in human datasets (Fig. 2B). The substantial number of somatic mutations observed in chimpanzee HCC samples, particularly indels, highlights potential differences in genomic stability and repair mechanisms between the two species. There were 10.5% more non-synonymous SNVs in humans compared with chimpanzees (61.7% non-synonymous in chimpanzee vs. 72.2% in human; Fisher’s exact test, *P* < 2e-16; Fig. 2C; Supplementary Table S10). This result could imply differences in the evolutionary pressures faced by humans and chimpanzee HCC genomes, with either stronger positive selection or weaker purifying selection operating on HCC-related genes in humans relative to chimpanzees.

#### TSC2 mutations and associated somatic mutation profiles

*TSC2* functions as a tumor suppressor and is a critical negative regulator of the pro-oncogenic mTOR signaling pathway [59–61]. Eight non-synonymous somatic mutations were identified in *TSC2* across chimpanzee HCC samples, including five SNVs, two deletions, and one insertion (Supplementary Table S11). None of these mutations were located at the same positions across different samples, indicating diverse mutation sites within this tumor suppressor gene. A similar pattern was observed in the human datasets. In the ICGC set, four mutations were identified in *TSC2*, comprising two SNVs, one insertion, and one deletion. In the TCGA+ set, six mutations in *TSC2* were identified, including five deletions and one SNV. As with the chimpanzee samples, none of these mutations were located at the same positions, further highlighting the variability in mutation sites within TSC2.

#### Shared somatic mutations between chimpanzee and human HCC

**Figure 3:**
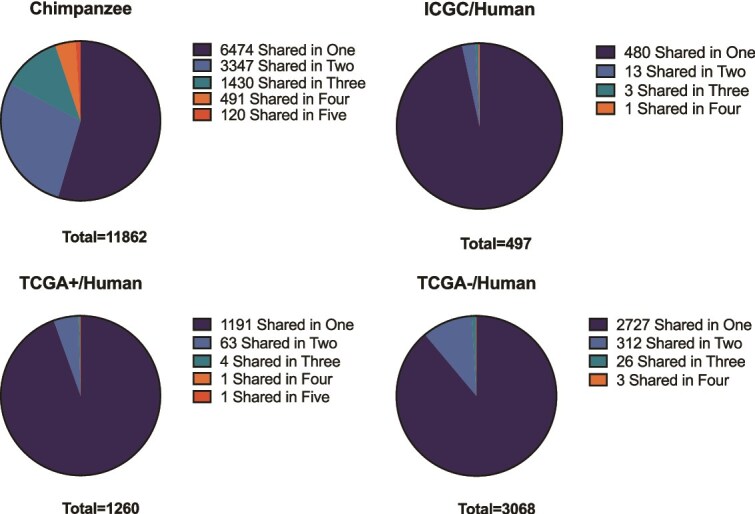
Gene-level overlap among samples with HCC-specific somatic mutations in chimpanzee and three human HCC datasets, ICGC, TCGA+ (with mutant TSC2), and TCGA+ (without mutant TSC2).

A total of 93 somatic mutations were shared between at least two out of five chimpanzee HCC samples (Fig. 3), including four genes with multiple shared mutations: *BAG6*, *BIVM-ERCC5*, *MTMR3*, and *NUP153* (Table 1). Due to the lower number of mutations in human datasets, no mutation was shared among human samples. At the gene level, chimpanzee samples exhibited higher overlaps than human datasets, likely due to the higher number of mutations found in chimpanzee HCC samples (Fig. 3).

**Table 1:** Genes with multiple shared mutations in chimpanzee HCC samples.

Gene	Mutations	AminoAcidChange	Samples	GeneDescription
BAG6	1879A > G; 1895C > T	Ala627Thr; Thr632Met	S2, S4	BAG Cochaperone 6
BIVM-ERCC5	2374 T > A; 4600G > C	Ser792Thr; Gly1534Arg	S2, S3; S3, S5	Read-through transcription between the neighboring BIVM and ERCC5
MTMR3	2411A > G; 2524A > G	Asp804Gly; Met842Val	S3, S5	Myotubularin-related protein 3
NUP153	3158C > T; 3148 T > C	Thr1053Ile; Phe1050Leu	S3, S5	Nucleoporin 153

**Figure 4:**
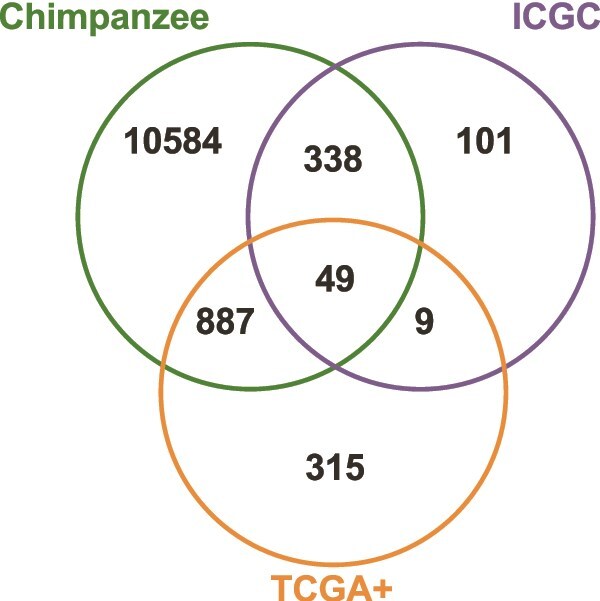
Numbers of genes with HCC-specific somatic mutations, shared between chimpanzee two human datasets (ICGC and TCGA+).

To investigate overlaps between chimpanzee and human HCC samples, we included only the two human datasets with TSC2 mutations (ICGC & TCGA+). In addition to *TSC2*, we found 48 other genes with mutations present in chimpanzee and both human HCC datasets, including *TTN, FAT4, TP53, SPEN, LRP1B*, and *STAG1* (Fig. 4; Table 2; the full list of overlapping mutant genes is provided in Supplementary Table S12). The comparative analysis of somatic mutations in chimpanzees and humans reveals distinct mutation patterns, with chimpanzees exhibiting a higher proportion of indels. The presence of shared mutations and genes between species, particularly in genes implicated in cancer-related processes, underscores potential conserved mechanisms in HCC pathogenesis.

**Table 2:** Mutant genes shared among chimpanzee samples and human TSC2-positive datasets (TCGA+ and ICGC). The number indicates the count of samples in each dataset with HCC-specific mutations within each gene.

Gene	Chimpanzee	TCGA+	ICGC	GeneDescription
TSC2	4	5	4	Tuberous sclerosis complex 2
TTN	5	4	1	Titin
FAT4	5	1	1	FAT Atypical Cadherin 4
HYDIN	4	2	1	HYDIN Axonemal Central Pair Apparatus Protein
MYOM1	5	1	1	Myomesin 1
PCDHGA4	4	2	1	Protocadherin Gamma Subfamily A, 4
SPEN	5	1	1	Spen Family Transcriptional Repressor
TP53	2	2	3	Tumor Protein P53
UBR4	4	2	1	Ubiquitin Protein Ligase E3 Component N-Recognin 4
ALMS1	4	1	1	ALMS1 Centrosome And Basal Body Associated Protein
ANK2	4	1	1	Ankyrin 2
BDP1	4	1	1	B Double Prime 1
ENSPTRG00000044766	4	1	1	–
LRP1B	4	1	1	LDL Receptor Related Protein 1B
NCAPD3	3	1	2	Non-SMC Condensin II Complex Subunit D3
STAG1	4	1	1	STAG1 Cohesin Complex Component
UTRN	4	1	1	Utrophin

To gain deeper insights into the mutational landscape and potential conservation of oncogenic drivers between chimpanzee and human HCC, we examined mutations in chimpanzee samples within a set of 23 recently identified HCC coding driver genes derived from a comprehensive analysis of 494 human HCC samples [62]. Among these 23 coding driver genes, 18 harbored mutations in at least one chimpanzee HCC sample (Supplementary Table S13). Beyond *TSC2*, which has been implicated in HCC subtype differentiation, we identified three additional driver genes—*ARID1A*, *BRD7*, and *FGA*—that were recurrently mutated in four chimpanzee HCC samples.

### Microsatellite instability in chimpanzee and human HCC

**Figure 5:**
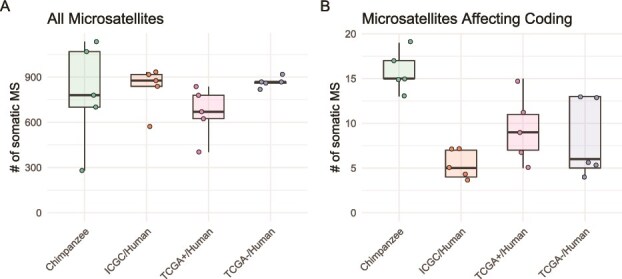
Comparison of SMVs among chimpanzee HCC samples and three human HCC datasets, with all microsatellites (A) or microsatellites within coding region (B).

Somatic microsatellite variants (SMVs) in cancer are indicative of microsatellite instability, often resulting from defects in the DNA mismatch repair system. On average, 793 SMVs were found in chimpanzee HCC samples (range: 280–1135), compared to 827, 662, 866 SMVs found in ICGC, TCGA+, and TCGA- datasets, respectively (Fig. 5A). A slightly higher proportion of coding-affecting SMVs was found in chimpanzee samples (2%, Fig. 5B) compared with human samples (1%, Fig. 5B).

## DISCUSSION

HCC, the most common form of liver cancer, is genetically diverse and histologically variable, leading to its classification into several subtypes. The most common subtype, classical HCC subtype, frequently involves mutations in *CTNNB1, TERT, TP53*, and *ARID1A* genes [63]. Fibrinogen alpha chain (*FGA*) has been determined to regulate HCC progression and metastasis [62]. In the rare and aggressive Scirrhous-HCC subtype, which is characterized by dense fibrous stroma and small tumor nests, frequent Tuberous Sclerosis Complex 1 (*TSC1*)/Tuberous Sclerosis Complex 2 (*TSC2*) mutations have been prevalent [57]. Interestingly, *TSC2* alterations are present in only 7% of human HCC patients [64], whereas 80% of chimpanzees with HCC in our study possessed *TSC2* mutations. *TSC2* is a tumor suppressor gene that encodes the protein Tuberin complexing with Hamartin, the protein encoded by *TSC1*, and together they inhibit the mammalian target of rapamycin (mTOR) pathway, which is crucial for cell growth, metabolism, and proliferation. Clinical trials are currently testing the efficacy of mTOR inhibitors in HCC patients with *TSC1*/*TSC2* alterations [65]. Variants in *TSC2* are associated with Tuberous Sclerosis Complex, a condition characterized by developmental issues and the growth of benign tumors in multiple body parts. Alterations in *TSC2* have been identified in 3.39% of all cancers, with a higher prevalence in lung adenocarcinoma, colon adenocarcinoma, breast invasive ductal carcinoma, endometrial endometrioid adenocarcinoma, and high-grade ovarian serous adenocarcinoma [65].

Dysregulated Wnt/β-catenin signaling is implicated in ~40% of HCC cases [66]. This pathway’s aberrant activation, often due to mutations in components like *CTNNB1* (encoding β-catenin), plays a significant role in HCC pathogenesis. Notably, the tumor suppressor gene FAT Atypical Cadherin 4 (*FAT4*), which regulates Wnt/β-catenin signaling, is mutated in all HCC cases in our chimpanzee study. FAT family genes are frequently mutated across multiple human cancer types and also found in HCC [67]. In human HCC data collected from 379 patients in the TCGA database, alterations in FAT family genes occur with the following frequencies: *FAT4* in 5.74%, FAT Atypical Cadherin 2 (*FAT2*) in 5.46%, FAT Atypical Cadherin 1 (*FAT1*) in 6.28%, and FAT Atypical Cadherin 3 (*FAT3*) in 7.38% of cases [68]. In our study, we found that *FAT2* was mutated in all chimpanzee HCC samples, *FAT1* and *FAT3* were mutated in 4 out of 5 samples. Of these FAT family genes, *FAT4* has the strongest association to HCC [69] and these mutations likely contribute to the high expression of *CTNNB1* observed in the immunohistochemistry results and play a vital role in HCC initiation or progression.

Another gene of interest we discovered with a strong association to HCC, which was altered in two chimpanzee samples, is *TP53*. Known as the “guardian of the genome,” *TP53* regulates the cell cycle via checkpoints, induces cell cycle arrest when damage is detected, and can promote apoptosis. Beyond its central role in human cancer, *TP53* has also been a focus of comparative oncology: elephants, for instance, have evolved up to 20 functional copies of *TP53*, a genomic expansion thought to underlie their remarkable cancer resistance despite large body size and long lifespan [4, 5]. *TP53* is the most frequently mutated gene in human cancers [70] and has been shown to be altered in 32% of human HCC cases [68]. Some studies suggest that the two most frequently mutated genes in HCC, *TP53,* and *CTNNB1*, occur mostly exclusively [71, 72].

Not only does variation in cancer genome heterogeneity among species remain unknown, but the patterns of genomic heterogeneity and selective pressures underlying them across various cancer types in humans are also poorly understood [73]. Human HCC-related genes are characterized by relatively high dN/dS values relative to other cancer types, exceeded only by pancreatic carcinoma, suggesting increased levels of adaptive evolution [73]. While the SNV heterogeneity level in chimpanzee HCC is at least an order of magnitude higher, the signal of positive selection measured by non-synonymous SNVs is 10.5% stronger in humans. The observed differences in stability and potentially stronger positive selection pressures on human HCC genome may imply that evolutionarily HCC has been an earlier genomic challenge for humans than for chimpanzees. Chronic hepatitis B causes almost 40% of HCC in humans, and ancient HBV sequences have been found in fossilized remains of humans dating back to the Neolithic period around 7000 years ago [74]. Many ape species harbor endogenous HBV strains, but no evidence exists to suggest that these strains can infect humans [75, 76], implying a higher virulence of human HBV strains. We also note that while our dataset indicates a greater non-synonymous excess in human compared with chimpanzee HCCs, we did not perform formal gene- or site-level evolutionary modeling (such as dNdScv [77] or cancereffectsizeR [78]) owing to the small chimpanzee cohort and thus limited statistical power. Future work with expanded chimpanzee cohorts and deeper mutation datasets will be required to apply robust selection-inference models and validate inter-species differences in evolutionary pressure.

The unique genetic alterations identified in the chimpanzee HCC genomes, particularly the high prevalence of *TSC2* mutations, suggest species-specific pathways of oncogenesis. The role of *TSC2* and the mTOR signaling pathway in cancer progression is well-established in humans, and the high frequency of these mutations in chimpanzee samples may indicate a parallel, yet distinct, evolutionary adaptation in this species. This finding opens up new avenues for research into how similar oncogenic pathways may be differentially regulated across species and how these differences could inform targeted therapies. Understanding these variations could lead to the identification of novel therapeutic targets and biomarkers that are not apparent when focusing solely on human cancer genomics.

To address a possibility that the elevated somatic mutation burden observed in chimpanzee tumors is attributable to formalin-fixed paraffin-embedded (FFPE)-related artifacts rather than innate causes, we implemented a more stringent variant filtering pipeline (applied through FilterMutectCalls; see Materials and Methods) specifically designed to mitigate known sources of FFPE-induced noise, resulting in a high-confidence variants set (Chimpanzee-HQ). Even after applying these stringent filters, chimpanzee tumors retained a substantially higher number of somatic mutations (average of 1742) compared to human tumors (Supplementary Fig. S6). Within the human cohort, mutation counts did not substantially differ between FFPE-preserved (TCGA+) and non-FFPE samples (Supplementary Table S4), suggesting that fixation method alone does not account for the observed interspecies disparity. We recognize that FFPE preservation can introduce context-dependent artifacts, particularly C > T transitions arising from cytosine deamination and low-frequency variants near read ends. Although the stringent filtering substantially reduced the overall variant count, the qualitative mutational spectrum remained consistent (Supplementary Fig. S3A and B), supporting the interpretation that the remaining variants likely reflect underlying biological processes rather than purely technical noise. Yet, we acknowledge that FFPE-associated effects cannot be entirely excluded, particularly in the absence of matched fresh-frozen chimpanzee tumor samples. The current data indicate that the elevated mutational burden in chimpanzee HCC cannot be fully attributed to FFPE effects and likely reflects true differences in tumor evolution and genome instability between species.

The chimpanzee reference genome and catalog of population polymorphisms remain less comprehensive than their human counterparts, which may result in some residual germline variants to be misclassified as somatic mutations, despite the use of a panel of normals (PoN) derived from a larger chimpanzee population. Although the mutational spectrum remained qualitatively stable after stringent filtering (Supplementary Fig. S3), we cannot entirely exclude the possibility that a fraction of germline variants or reference-related artifacts contributed to the elevated mutation burden. Nevertheless, the magnitude of the difference in mutation counts between chimpanzee and human HCCs, even under conservative assumptions and rigorous filtering, suggests that these technical factors alone are unlikely to fully account for the observed disparity. We therefore interpret this as evidence of a genuine increase in somatic mutation burden in chimpanzee HCC.

It is tempting to speculate that the observed divergence in mutational patterns between chimpanzee and human HCCs likely reflects broader life-history and evolutionary differences between the two species. Humans differ from chimpanzees in several key life-history traits, including markedly longer lifespan, delayed sexual maturity, higher birth weights, longer gestation, slower juvenile growth, and an extended post-reproductive lifespan [79]. An adolescent growth spurt, long thought to be unique to humans, seems to occur in chimpanzees as well [80]. Chimpanzees, in contrast, mature earlier, experience higher adult mortality, and reproduce more frequently but have longer mother–infant dependency periods. These contrasts may contribute to distinct evolutionary trade-offs between somatic maintenance, reproductive output, and cancer susceptibility. In humans, extended longevity and delayed maturity intensify selection for genome maintenance and tumor suppression over longer timescales, whereas in chimpanzees, faster turnover and shorter lifespan may favor more transient but effective early-life defenses. Human fitness is further decoupled from longevity by post-reproductive survival and cooperative care, while chimpanzees forfeit potential lifetime fertility through adult mortality attrition [79]. Variation in infant mortality and fertility rates drives demographic differences between species, underscoring that cancer resistance, too, evolves within these same life-history constraints.

However, we also recognize that the small cohort size (*n* = 5) used in our study limits generalizability. As such, we view this study as exploratory and hypothesis-generating. Access to chimpanzee materials is increasingly limited, as these animals are retired from biomedical research and naturally exhibit relatively low cancer incidence. Consequently, future studies—where feasible—with larger chimpanzee cohorts and expanded germline variant resources will be required to validate these findings and to further clarify species-specific differences in mutational processes and selective pressures between humans and other primates.

## Supplementary Material

eoaf038_supplement_files

## Data Availability

All sequence data have been deposited at NCBI SRA with accession # PRJNA1150607.
