# Supplementary figures and images for: Divergent evolution of hepatocellular carcinoma genomes in chimpanzees and humans
Source: Evol Med Public Health. 2025 Dec 15;14(1):1–14. doi: 10.1093/emph/eoaf038 (PMC12783088; doi:10.1093/emph/eoaf038)

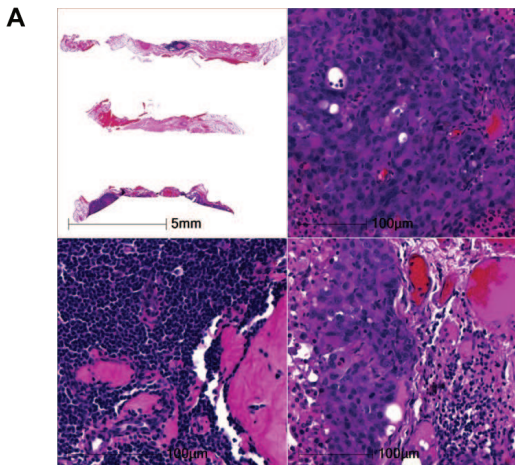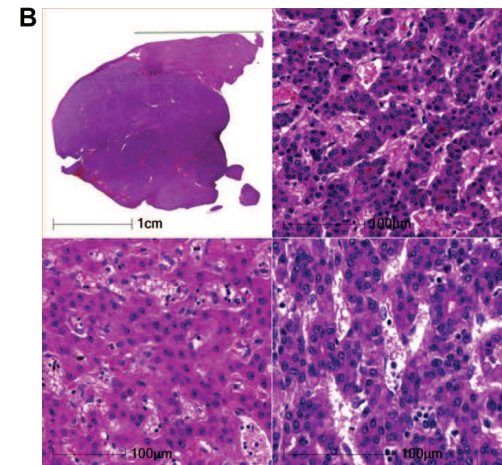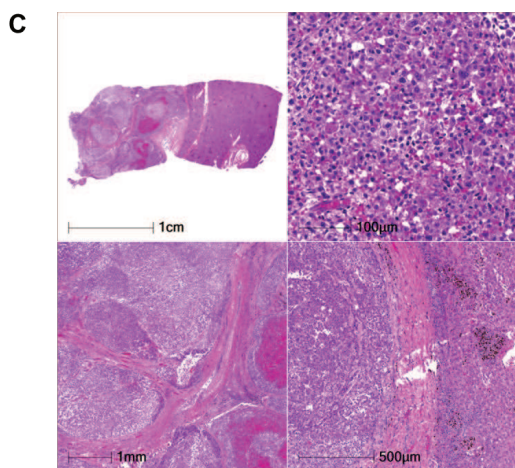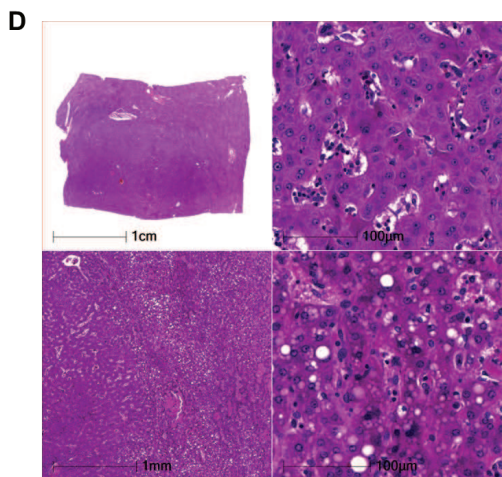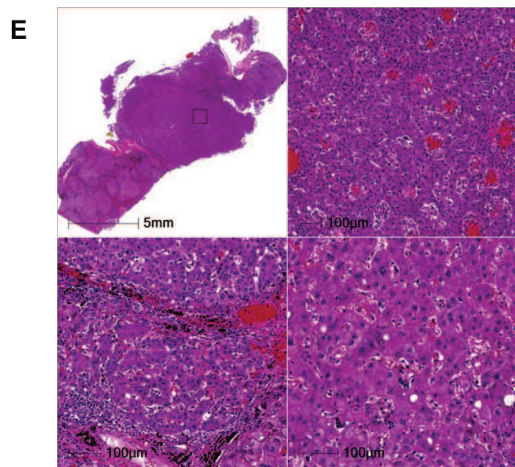

Supplement: eoaf038_supplement_files [file eoaf038_supplement_files.zip › eoaf038_supplement files/FigS1new.cmyk.pdf]

**A**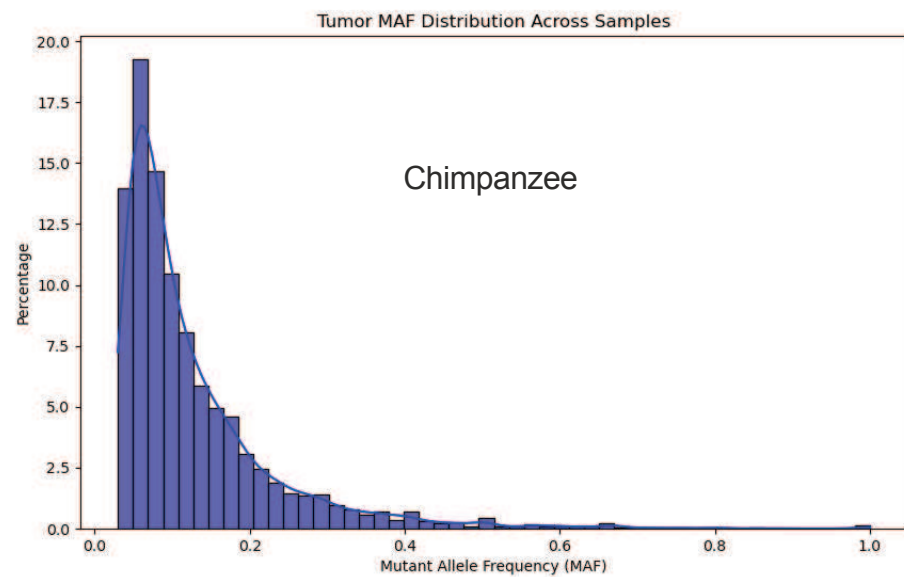**B**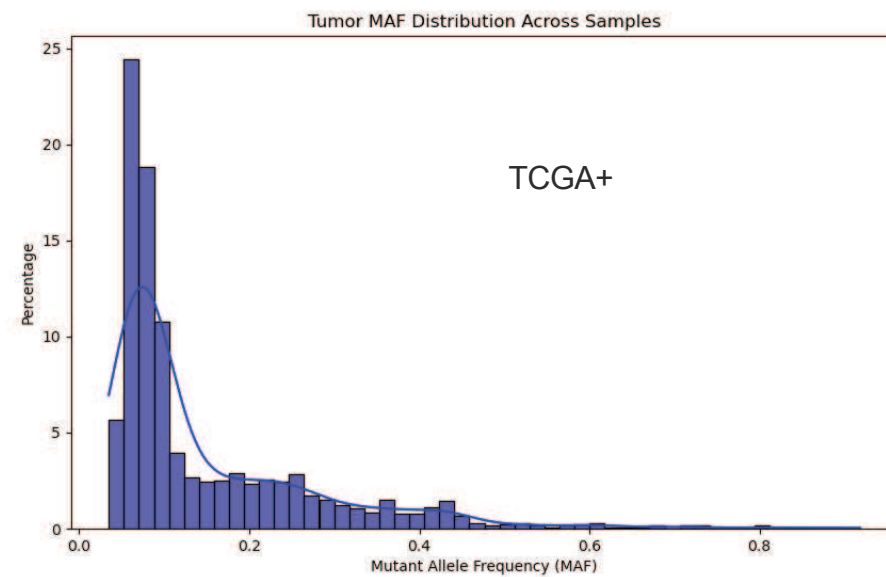**C**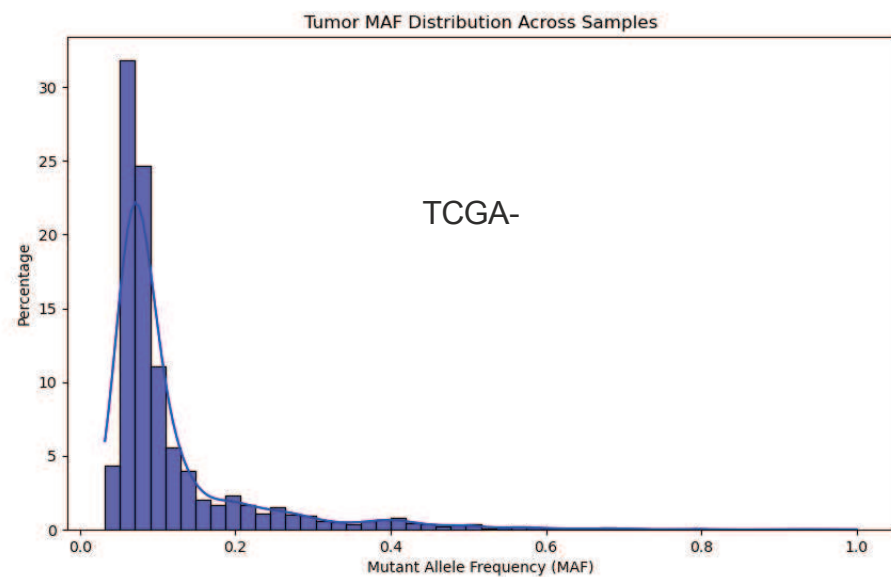**D**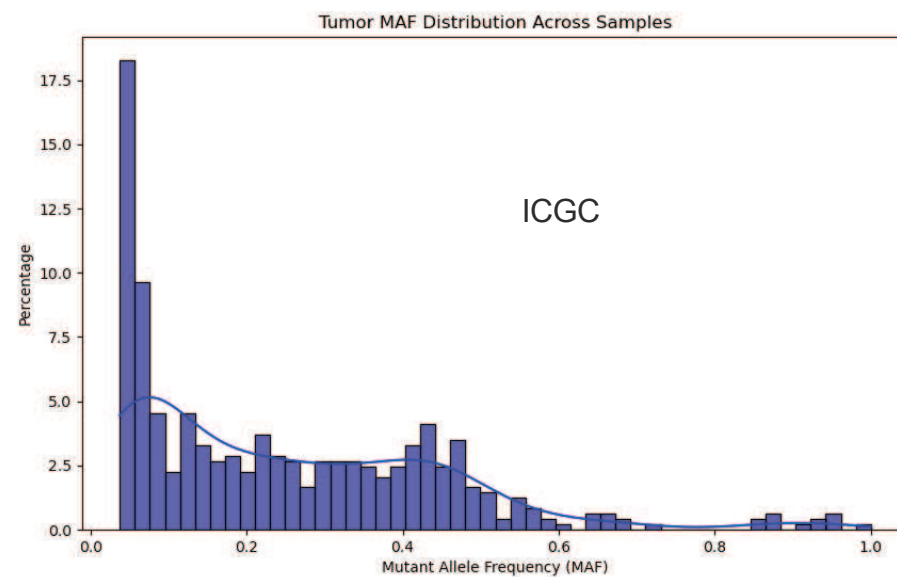

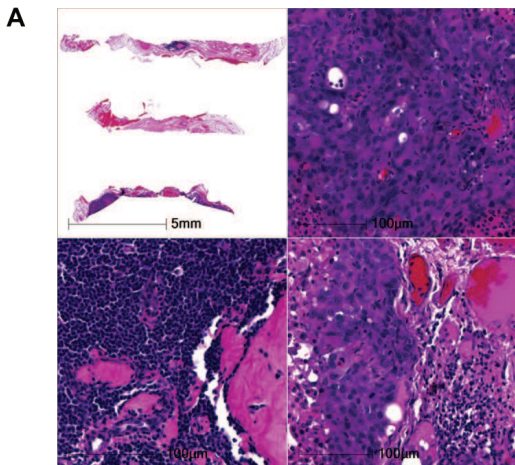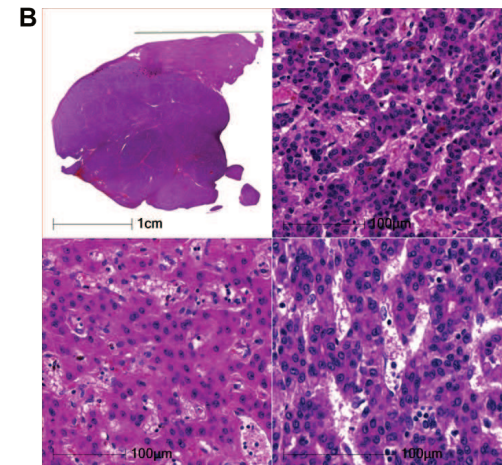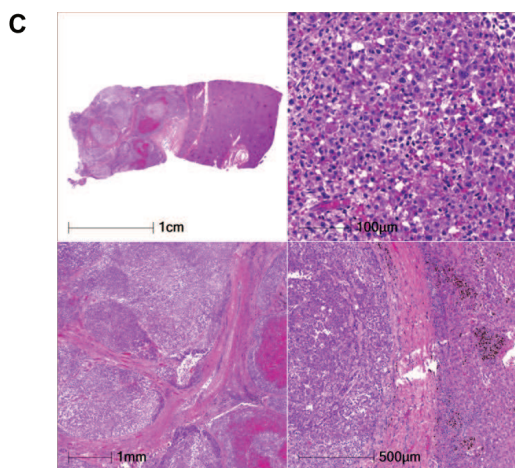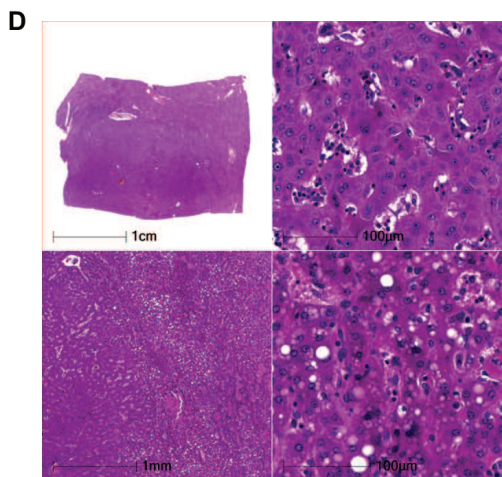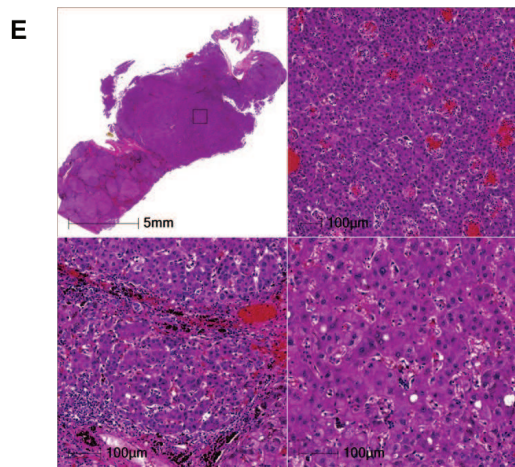

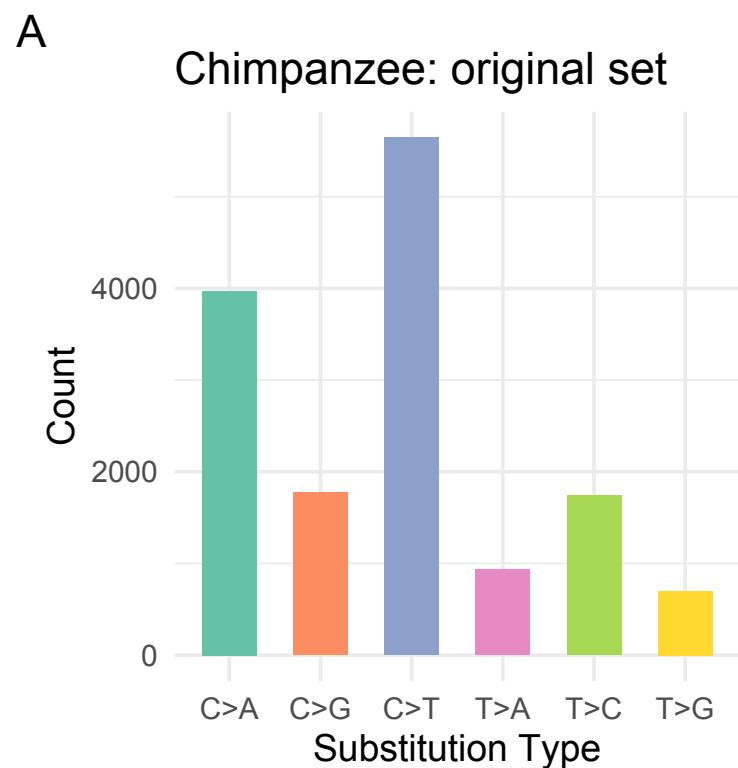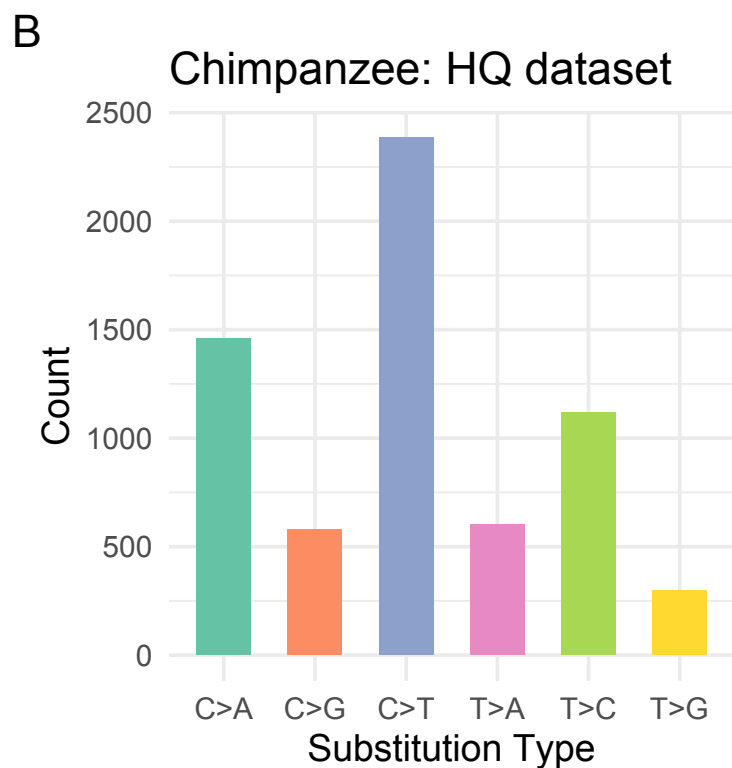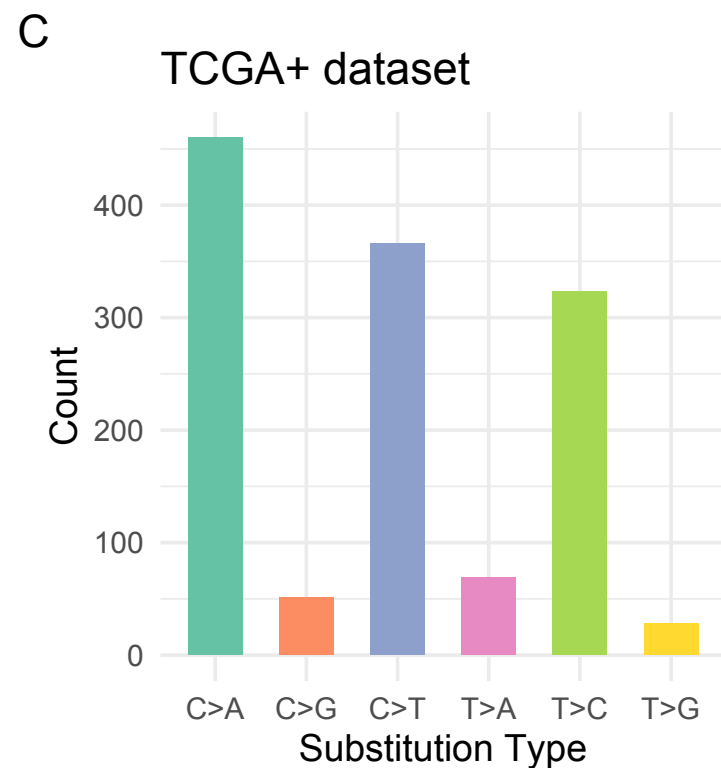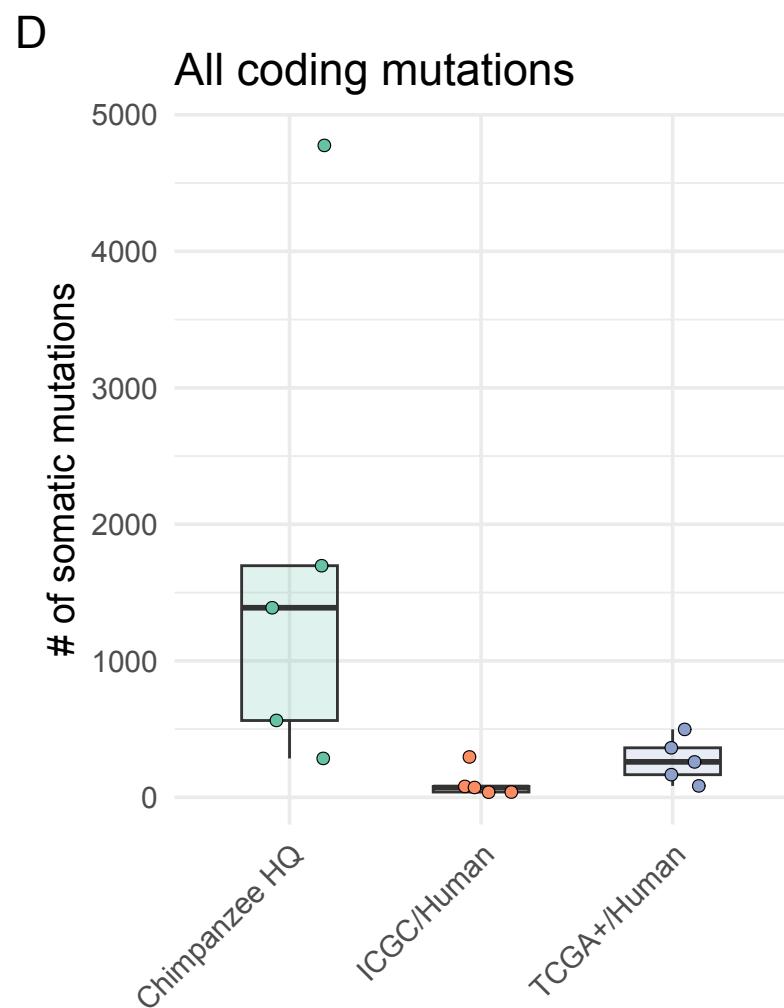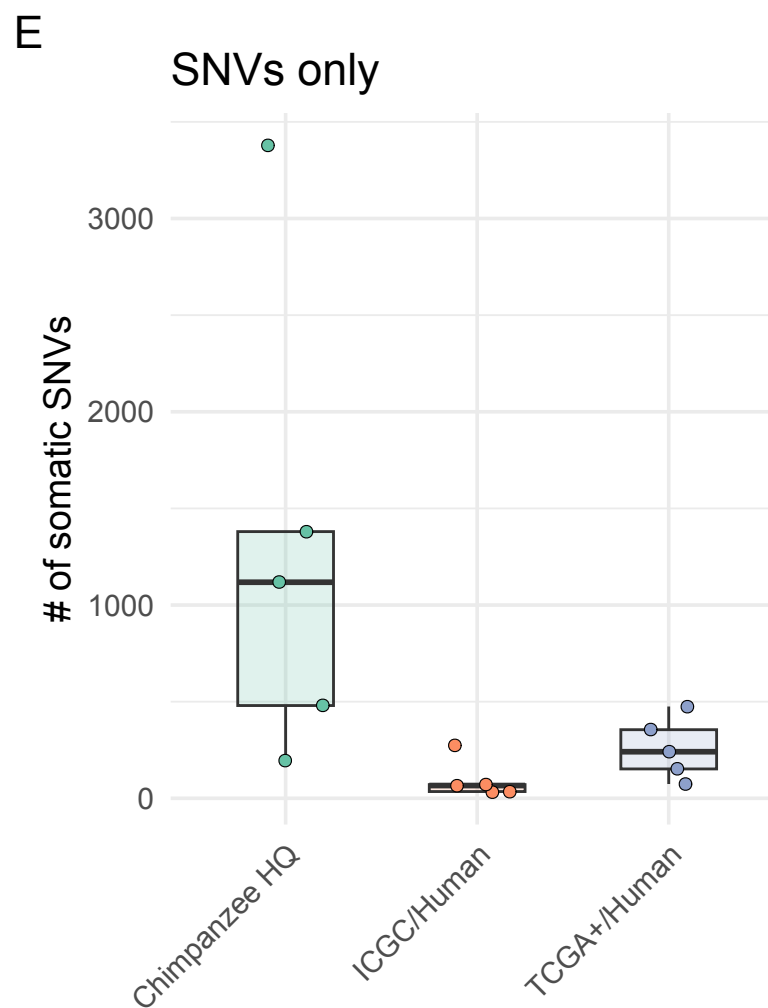

Supplement: eoaf038_supplement_files [file eoaf038_supplement_files.zip › eoaf038_supplement files/FigS2new.cmyk.pdf]
